# ACURATE *neo*™ aortic valve implantation via carotid artery access: first case report

**DOI:** 10.1186/s13019-021-01544-5

**Published:** 2021-06-04

**Authors:** Chadi Aludaat, Alexandre Canville, Quentin Landolff, Matthieu Godin, Fabrice Bauer, Fabien Doguet

**Affiliations:** 1 Department of Cardiac Surgery, Heart Team Unit, Rouen University Hospital, Hôpital Charles Nicolle, F76000 Rouen, France; 2 Department of Cardiology, Clinique St. Hilaire, Heart Team Unit, F76000 Rouen, France

**Keywords:** TAVR, Carotid artery access, Case report

## Abstract

**Background:**

The ACURATE *neo*™ transcatheter heart valve (Boston Scientific, Marlborough, Massachusetts) is predominantly implanted via femoral access. Transcarotid use of this prothesis has never been reported.

**Case presentation:**

We present the case of an 89-year-old woman referred to us for a transcatheter aortic valve replacement (TAVR). After apparatus imaging of the aortic annulus and the peripheral vascular pathway, the heart team was confronted with a triple challenge: (i) The preferable choice of a self-expanding valve because of a small aortic annulus in an obese woman. (ii) Gaining favorable access to the coronary ostia, considering multiple recent coronary stenting. (iii) Utilizing an alternative arterial access because of iliac and femoral severely calcified stenosis. Implanting the ACURATE *neo*™ transcatheter heart valve (THV) via carotidal access allowed us to overcome these challenges. The procedure was performed successfully without any short-term complications.

**Conclusion:**

We report the first case of implantation of an ACURATE *neo*™ transcatheter heart valve (Boston Scientific, Marlborough, Massachusetts) via the right common carotid artery.

## Background

In patients ineligible for transcatheter aortic valve replacement (TAVR) via the traditional femoral access route due to severe peripheral vascular disease, carotid artery access is a suitable alternative thoracic access route [1, 2]. While Self-expanding CoreValve® (Medtronic, Minneapolis, MN, USA) were the first transcatheter heart valves (THV) to be implanted via this non-femoral access [2] Here, we report the first implantation of an ACURATE *neo*™ aortic valve system via the right common carotid artery access (RCCA).

## Case report

**Fig. 1:**
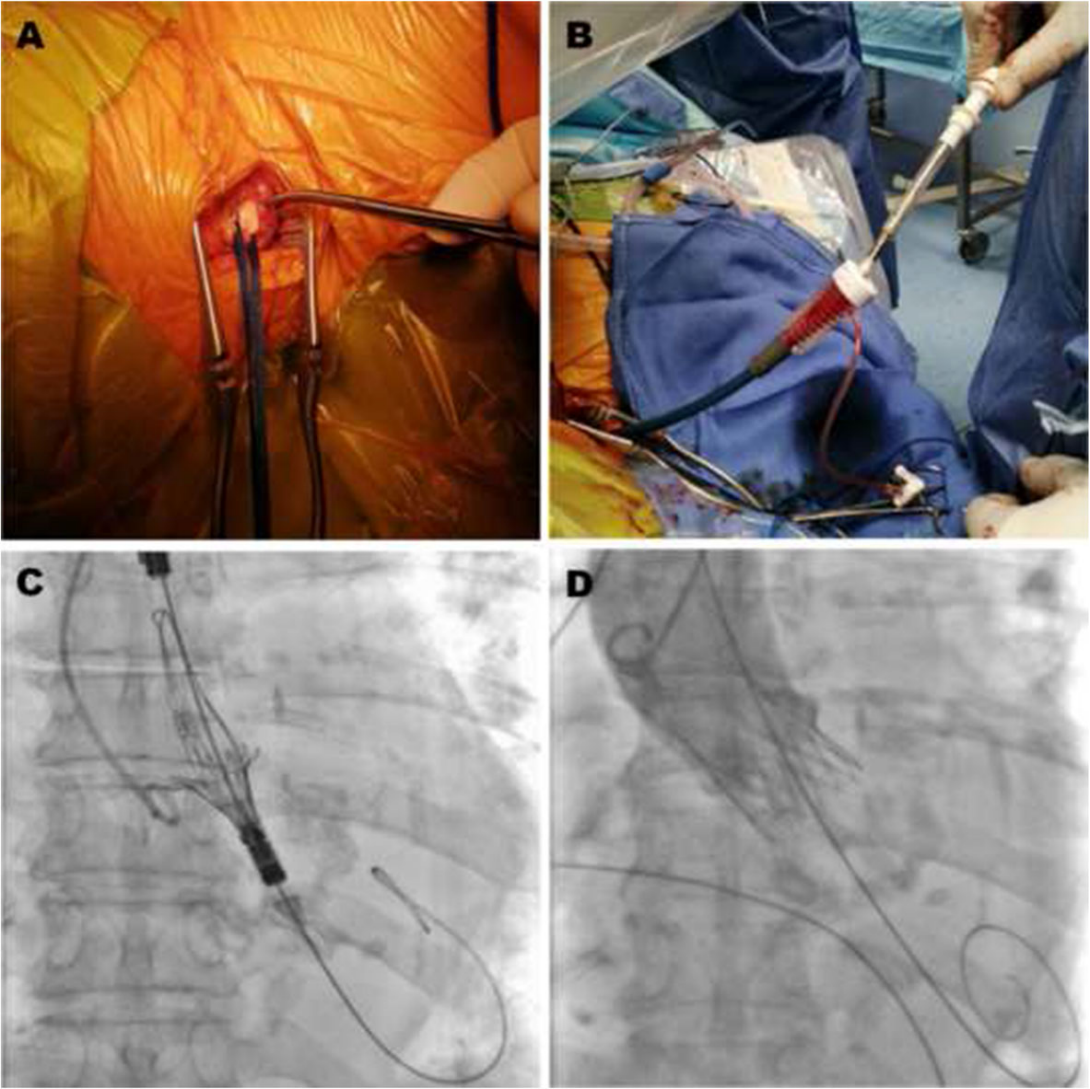
Cervical cut down and right common carotid artery (RCCA) exposed surgically (**A**); ACURATE™ neo advancement into the iSLEEVE™ through the RCCA (**B**) Top to down ACURATE neo deployment (**C**) Final aortography showing no paravalvular leak (**D**)

An 89-year-old female patient requiring surgery for colorectal cancer also suffered from severe calcified aortic valve stenosis (aortic mean gradient = 59 mmHg, aortic area = 0.4 cm^2^) and significant stenosis of the left main trunk, which contraindicated her cancer surgery. Discussions among the local heart team led to suggest that percutaneous coronary intervention followed by TAVR would be performed before colorectal surgery. Considering future concern of coronary intervention, easy access to the coronary ostia is expected for selection of the THV. Aortic root dimensions and the access site were precisely measured using computed tomography (CT) angiography, with the aortic annulus having a perimeter of 67 mm and an area of 320 mm^2^. While iliac and femoral severely calcified stenosis contraindicate a conventional femoral route, the Doppler and CT studies of supra-aortic vessels did not show any abnormalities. The right common carotid artery was free from calcifications, and its internal diameter was 7 mm at puncture level. The implantation of an S-size ACURATE *neo*™ aortic valve (Boston Scientific, MA, USA) via the right carotid artery was collegially retained by the local heart team. The choice of this valve was mainly driven by an easy access to the coronary ostia if repeated percutaneous revascularization were requested in the future in this coronary diseased patient. After obtaining the patient’s consent, the TAVR procedure was performed according to local standard protocol in a hybrid operating room. Intravenous heparin was administrated to maintain an activated clotting time of ≥250 s. As in our usual practice for transcarotid TAVR, continuous cerebral perfusion was monitored by cerebral tissue oxygen saturation measurement with the near infrared spectroscopy (NIRS) cerebral oximeter (O3™, Masimo, Irvine, CA). The patient was under double anti-platelet therapy for a recent previous coronary stent implantation. Surgical access for TAVR was performed under general anesthesia with a fast-track approach. A 5 Fr pigtail was advanced into the aortic root using a left radial approach. Through a 4 cm right cervical incision (Fig. 1A), the right common artery was exposed. The stenotic aortic valve was then crossed using a straight-tip guide wire and an Amplats left 1.0 diagnostic catheter (Terumo Medical, Somerset, NJ). The straight-tip guidewire was then replaced with a pre-shaped extra-stiff Safari XS (Boston Scientific, MA, USA) in the left ventricle. An iSLEEVE™ (Boston Scientific, MA, USA) expandable sheet was cautiously advanced into the ascending aorta (Fig. 1B). Given the heavy annular calcification, we performed a 22 mm valvuloplasty using a noncompliant balloon (Cristal balloon, BALT, Montmorency, France). Prothesis placement was performed in the standard fashion, and the up-to-down deployment was satisfactory with enhanced stability because of the short distance between the arterial carotid access and the aortic annulus (Fig. 1C). After a satisfactory angiogram (Fig. 1D), the iSLEEVE™ introducer was removed, and the common carotid artery was surgically purged and repaired using a 6–0 polypropylene running suture. Echocardiography confirmed the absence of paravalvular leak, and the mean prosthetic gradient was 7 mmHg. After continuous ECG monitoring, the patient was discharged home on the third postoperative day, per our local practice. Based on the Valve Academic Research Consortium (VARC 2) criteria, we recorded no complications.

## Discussion

We describe the first implantation of an ACURATE *neo*™ aortic valve via right carotid artery access, which was performed successfully, without short-term complications. According to the current medical literature, 10 to 15% of patients are still ineligible for the transfemoral route despite technical advances in reductions to the valve introducer size [2, 3]. Transapical and direct aortic TAVR were initially attempted, but since the first transcarotid TAVR in Lille, France, in 2010 [4], this alternative access for TAVR has emerged as a reliable alternative to the more traditional non-femoral access. Data from large registries demonstrate favorable outcomes with a high rate of procedural success and low 30-day and 1-year mortality with the transcarotid route [5]. Like other valves previously implanted via carotid artery access, the CoreValve® Evolut™ R (Medtronic, Minneapolis, MN, USA) and SAPIEN 3 (Edwards Lifesciences, Irvine, CA, USA) have been validated for these indications, demonstrating their non-inferiority compared to conventional use in a percutaneous femoral arterial route [5]. In our heart team unit, the carotid approach is the first choice among alternative non-femoral approaches for TAVR. During the procedure, we did not encounter any issue related to the handling of the iSLEEVE™ introduced into the RCCA or strict valve positioning. The ACURATE *neo*™ (Symetis/Boston, Ecublens, Switzerland) is a new-generation self-expanding device characterized by an X-shaped stent design with a unique deployment mechanism [6]. ACURATE *neo*™ demonstrated improved procedural outcomes, lower bleeding complications and reduced post-operative length of stay [5]. In terms of carotid access, self-expandable valves are safe, meaning no specific preprocedural imaging were performed regardless CT scan and Doppler echocardiography. However, because nothing is known about the ACURATE *neo*™ when used through the carotid route, we paid attention to closely monitor per-procedural cerebral perfusion with the use of near infrared spectroscopy. But above all conceptually, this supra-annular self-expanding THVs may mitigate the risk of coronary obstruction, furthermore, coronary re-access may be less challenging because of the short stent body and the open-cell design of the upper crown as compared to the other self-expanding THVs, particularly the Evolut™ R (Medtronic, Minneapolis, MN, USA) [6]. Patients at risk of future percutaneous coronary interventions may be suitable candidate for this prothesis [6].

## Conclusion

We report the first case of an aortic ACURATE *neo*™ aortic valve system implantation via right carotid artery access. With the expanding indications of TAVR, further studies are required to assess this self-expandable valve as a suitable option for the treatment of aortic stenosis via carotid artery access particularly for those patients necessitating future coronary revascularization and contraindicated for the transfemoral approach.

## Data Availability

Not applicable.

## Abbreviations

RCCA: Right common carotid artery
TAVR: Trans aortic valve replacement
ECG: Electrocardiogram
